# A scoping review protocol of the rehabilitation needs of people with brain tumours

**DOI:** 10.12688/hrbopenres.13773.1

**Published:** 2023-09-07

**Authors:** B. O'Donovan, A. Malone, F. Horgan, K. Bennett

**Affiliations:** 1 School of Population Health, RCSI University of Medicine and Health Sciences, Dublin, Ireland; 2 School of Physiotherapy, RCSI University of Medicine and Health Sciences, Dublin, Ireland

**Keywords:** Rehabilitation, Brain tumour, Physical therapy, Occupational therapy, Interventions, Scoping review, Study protocol

## Abstract

**Background:** Every year 480 people are diagnosed with a primary brain tumour in Ireland. Brain tumours can vary in type, location, treatment, and progression but neurological impairments are a consistent feature. Such neurological disability creates significant symptom burden that can seriously impact peoples’ functional ability and quality of life. Rehabilitation can improve functional prognosis (motor and cognitive) and quality of life in people with brain tumours. However, research and experience consistently show that people with brain tumours can have difficulties accessing rehabilitation services. Our scoping review will investigate the research evidence concerning the rehabilitation needs of people with brain tumours.

**Methods:** The scoping review will be conducted in line with Preferred Reporting Items for Systematic reviews and Meta-Analyses extension for Scoping Reviews (PRISMA-ScR) reporting guidelines. Relevant databases (PubMed, Embase, CINAHL+, PsychINFO, PEDro) and grey literature sources will be searched. Publications relating to international rehabilitation practices will be included. A data extraction table will be created to facilitate narrative synthesis of the results.

**Conclusions:** This scoping review will examine the scope of the literature on the rehabilitation needs of people with brain tumours. The findings will inform a research project entitled “Surgery, radiotherapy, chemotherapy, but now what? Exploring the rehabilitation needs of people with brain tumours in Ireland”. An article reporting the results of the scoping review will be submitted to a scientific journal and presented at relevant national and international conferences.

## Introduction

In Ireland an average of 480 people are diagnosed with a primary brain tumour every year ^1^. A large number of people with other cancers—estimated at 9%—develop secondary brain tumours (metastases) ^2^. In general brain and central nervous system (CNS) tumours have a younger age-profile than many common cancers—median age at diagnosis for all Irish brain and CNS patients was 58 years from 1994–2013 ^1^. Brain tumours can shorten lifespan and create significant symptom burden as a result of neurological impairment ^3^. Symptoms can impact on the quality of life of brain tumour survivors—impacting people’s ability to work/travel, contributing to relational strain, depression, anxiety and increasing financial concerns ^4^.

Rehabilitation is a process that seeks to optimise patients’ independence and quality of life. Core values of rehabilitation practice include holistic collaboration within multi-disciplinary teams, collaborative goal-setting as well as education/training for patients and families. Physical/occupational therapy are frequently used to improve patients’ functioning in daily life and regain independence. Brain tumours present specific rehabilitation challenges. Early rehabilitation is important to employ neuroplasticity and reduce potential complications ^5^. However, the timing of rehabilitation is complicated by lengthy chemotherapy and/or radiotherapy treatment and side effects, leading to difficulty in determining the best time to emphasise rehabilitation. Furthermore, physical and cognitive impairments can fluctuate depending on whether the tumour responds to treatment or not, leading to difficulty with goal-setting around restorative versus adaptive rehabilitation. Given this heterogeneity and complexity, to date, there is a lack of evidence-based guidance on the rehabilitation needs and service use of people with brain tumours.

Nonetheless, some evidence has emerged that rehabilitation can improve functional prognosis (motor and cognitive) and quality of life in people with brain tumours ^6, 7^. A randomised controlled trial (RCT) in Denmark found significant within-group improvements to aerobic power and lower and upper limb strength following rehabilitation intervention ^8^. However, the small sample size (64 participants) was insufficient to determine superiority of the rehabilitation intervention compared to control. Research in acquired brain injury overall has shown that early access to rehabilitation can reduce potential complications, length of stay in hospital and improve patient outcomes ^5, 9^. Despite a general consensus on the therapeutic value of rehabilitation, it is recognised that there is significant lack of provision within existing neuro-rehabilitation services for people with brain tumours, who often experience difficulties accessing services ^5, 10^. Given the potential to benefit from rehabilitation, there is a need to synthesise current evidence to guide service provision and identify gaps for future research.

As part of the BRAIN-RESTORE programme of research, we aim to explore and understand the rehabilitation needs of people with brain tumours, to guide the development of effective rehabilitation services in Ireland. The BRAIN-RESTORE study has four interlinked work packages—a scoping review, cross sectional survey, epidemiological study and prospective study. The focus of this protocol is the scoping review which will review previous literature and research on rehabilitation needs of people with brain tumours.

### Aims/Objectives

The aim of this scoping review is to examine international evidence on rehabilitation needs of people with brain tumours and report rehabilitative interventions that have been investigated in the literature.

The specific objectives of the review are:

TEXT

1) To determine the extent and scope of current evidence on rehabilitation needs in people with brain tumours.
2) To chart the landscape of the types and range of interventions (physical and cognitive) for people with brain tumours.
3) To identify gaps in the literature for future research.

## Methods

Scoping studies are used to identify gaps in the existing literature and was selected as appropriate to meet the aims and objectives of this research ^11^. This scoping review was registered with the Open Science Framework ^12^. The overall conduct of the scoping review is informed by the JBI framework, while the Preferred Reporting Items for Systematic Reviews and Meta-analysis extension for scoping reviews (PRISMA-ScR) will be used to guide the reporting of the scoping review ^13^.

### Research question

A scoping review will be conducted to explore the question: What is known from empirical research about the rehabilitation needs of people with brain tumours and how these needs might be addressed?

### Study selection

The specific remit of the scoping review is to examine the rehabilitation needs of adult patients who were diagnosed with brain tumour. Full details of inclusion and exclusion criteria are presented below in Table 1. Inclusion criteria for studies are (i) adult patients (≥18 years); (ii) confirmed diagnosis of brain tumour according to WHO Classification of Central Nervous System tumours (iii) studies that examine the rehabilitation needs of people with primary/secondary (metastatic) brain tumours (iv) studies that describe patient outcomes related to rehabilitation interventions (physical and cognitive). Opinion pieces, editorials and commentaries will be excluded.

**Table 1.** Details of inclusion/exclusion criteria.

Criteria	Inclusion	Exclusion
Population	Aged 18 years or older Confirmed diagnosis of brain tumour (WHO 2021 Classification of Central Nervous System tumours (a. Glioma, glioneuronal and neuronal tumour, ependymoma; b. Cranial nerve tumour; c. Meningioma)) Primary/secondary brain tumours	Aged 17 years or younger
Intervention	Neuro-rehabilitation services	Not applicable
Outcomes	Any patient outcomes related to rehabilitation care Any patient outcomes related to rehabilitation interventions Physical needs *e.g.*, symptom burden, fatigue Cognitive needs *e.g.*, impaired memory/executive function Psychosocial needs *e.g.*, anxiety, depression, quality of life Resultant rehabilitation needs (physical & cognitive) Socioeconomic needs *e.g.*, financial burden Views of rehabilitation care – patients, HCP, carers, families.	Studies testing the psychometric properties of patient health measures.
Study Design	Studies with empirical research methods ( *e.g.*, randomized control trials, case control studies, cohort studies, and cross-sectional studies) Reviews	Non-empirical literature ( *e.g.*, opinion pieces, editorials)
Reporting	English language Sufficient detail on unmet rehabilitation needs Sufficient detail on results ^*^	Not applicable

*enough data reported in the results to be extracted from the paper and reported on in a meaningful manner according to our specified data extraction format. WHO, World Health Organization; HCP, healthcare personnel.

### Identifying relevant studies

The search strategy will be determined by the research team in consultation with a specialist librarian. Combinations of disease terms, rehabilitation terms and terms related to rehabilitation needs will be adapted from previous research ^7, 12^. A sample search strategy can be found in Table 2. Reference lists from papers of eligible studies and relevant journals will be checked to identify any potentially eligible articles that might have been missed by the electronic searches. The search will be conducted for research from 2003–2023 and will be restricted to English language papers only.

**Table 2.** Search terms and strategy for databases.

Databases 2003-2023	Search strategy
EMBASE PubMed CINAHL complete PsychINFO PEDro	(MH "Brain Neoplasms+") OR (Brain tumo* OR brain cancer* OR brain Metastas* OR brain neoplasm* OR malignant brain tumo* OR cerebral cancer* OR cerebral tumo* OR Glioblastoma multiforme OR Glioma* OR ((Brain OR Cranial) AND (tumo* OR Neoplasm OR Cancer))
	AND
	“rehabilitation” OR “physical therapy” OR “physical outcomes” OR “behaviour therapy” OR “behavior therapy” OR “cognitive therapy” OR “cognitive outcomes” OR “functional outcomes” OR “rehabilitation intervention”
	AND
	“unmet needs” OR “rehabilitation needs” OR “patient* needs” OR “rehabilitation assessment” OR “rehabilitation care needs” OR “quality of life” OR “symptom burden”

***Charting the data*.** At least two reviewers will independently screen titles and abstracts of records using reference management software, screening tools ( *e.g.*, COVIDENCE). Full text versions of potentially eligible papers will be examined to independently assess their suitability. Any disagreements will be solved by consensus or discussion by the research team. Data will be extracted on the following fields: (a) authors, study design, setting, year and country of study, data collection time period; (b) study population; (c) rehabilitation need investigated; (d) data collection method and instruments used, and (e) main results.

### Collating and summarising results 

A narrative synthesis of the data will be undertaken, with a structure based on: (i) the overall rehabilitation needs of people with brain tumours; (ii) evidence of unmet rehabilitation needs; (iii) interventions delivered. Where appropriate, illustrative quotes from study participants will be included. The results will be reported in the completed scoping review and presented in a PRISMA-ScR flowchart with a narrative summary.

## Discussion

This protocol describes the process and plans for a scoping review investigating the rehabilitation needs of people with brain tumours. It defines the objectives, methods, inclusion/exclusion criteria, data extraction and how results will be reported. The publication of the protocol is important in limiting reporting bias and may also be a useful tool for researchers preparing projects of evidence-synthesis related to rehabilitation needs or practices. Despite agreement among healthcare personnel (HCP) on the therapeutic value of rehabilitation, rehabilitation approaches for brain tumours are frequently lacking in clinical practice guidelines and “best current practice” is not defined ^13^. Brain tumour rehabilitation is complex with multiple challenges, however it is vital that the rehabilitation needs of patients are addressed, as rehabilitation services can lead to improved function and overall improvement in health-related quality of life. International evidence suggests that patients with primary brain tumours have unmet rehabilitation needs and a gap between patients’ needs and their use of multidisciplinary supports services can frequently exist ^8, 14– 18^. Evidence from this proposed scoping review has the potential to contribute to the wider literature and provide data on the impact of unmet needs on the lives of brain tumour survivors with limited/no access to rehabilitation services.

## Conclusion

This protocol presents our plans for a scoping review that will contribute to the evidence base of neuro-rehabilitation practices in Irish healthcare. The protocol describes the background and research methodology of a systematic research process. We consider findings emerging from the proposed scoping review will inform the development of evidence based, high quality rehabilitation services.

## Study status

The study has commenced (March 2023), with expected completion of the scoping review by November 2025.

## Dissemination

An article reporting the results of the scoping review will be submitted to a scientific journal and presented at relevant national and international conferences.

## Data Availability

No data are associated with this article.
