# Supplementary material for: A scoping review protocol of the rehabilitation needs of people with brain tumours
Source: HRB Open Res. 2024 Jan 29;6:45. Originally published 2023 Sep 7. [Version 2] doi: 10.12688/hrbopenres.13773.2 (PMC10831223; doi:10.12688/hrbopenres.13773.2)
Supplement: Peer review history

# Reviewer report: Reviewer response for version 2

**DOI:** 10.21956/hrbopenres.15196.r37917

**Published:** 2024-01-31

**Authors:** Emer Guinan

**Affiliations:** 1 School of Medicine, Trinity College Dublin, The University of Dublin Trinity College, Dublin, Leinster, Ireland

The authors have made changes to the manuscript in line with my recommendations. I have no further comments.

Is the study design appropriate for the research question?

Yes

Is the rationale for, and objectives of, the study clearly described?

Yes

Are sufficient details of the methods provided to allow replication by others?

Partly

Are the datasets clearly presented in a useable and accessible format?

Not applicable

Reviewer Expertise:

Cancer rehabilitation and survivorship

I confirm that I have read this submission and believe that I have an appropriate level of expertise to confirm that it is of an acceptable scientific standard.

# Reviewer report: Reviewer response for version 1

**DOI:** 10.21956/hrbopenres.15066.r36636

**Published:** 2023-11-16

**Authors:** Jennie Taylor, Melissa Brie

**Affiliations:** 1 Neurological Surgery, Division of Neuro-Oncology, University of California San Francisco, San Francisco, California, USA

This article provides a detailed plan of how their proposed scoping review will be performed in order to answer their research question about the rehabilitation needs of people with brain tumors. The results will be very helpful in further characterizing rehabilitation needs in this population and providing evidence to be able to advocate for services to address these needs. Objective 2 of the types of interventions being provided will hopefully inform strides toward a best practice approach.

 We would recommend including search terms of "cognitive rehabilitation," "speech therapy," "neurocognitive rehabilitation," "neuropsychological rehabilitation," "non-pharmacological intervention," "health behavior change" as these terms are often used in US clinical care and research and may yield results you would want to include in your review. While our program uses neuropsychologists to provide cognitive rehabilitation, of health system often uses speech therapists/speech language pathologists in that role. Also think it would be helpful to more clearly define rehab and address if there is longitudinal follow up in the interventions discussed as needs change over time, especially in this population.

Is the study design appropriate for the research question?

Yes

Is the rationale for, and objectives of, the study clearly described?

Yes

Are sufficient details of the methods provided to allow replication by others?

Yes

Are the datasets clearly presented in a useable and accessible format?

Not applicable

Reviewer Expertise:

Cognitive rehabilitation in primary brain tumor

We confirm that we have read this submission and believe that we have an appropriate level of expertise to confirm that it is of an acceptable scientific standard.

# Reviewer report: Reviewer response for version 1

**DOI:** 10.21956/hrbopenres.15066.r36120

**Published:** 2023-10-16

**Authors:** Emer Guinan

**Affiliations:** 1 School of Medicine, Trinity College Dublin, The University of Dublin Trinity College, Dublin, Leinster, Ireland

This scoping review proposes to examine the rehabilitation needs of people with brain tumours. The protocol is well written, clearly described and follows best practice frameworks for the conduct and proposed reporting of the review. Critically this area is completely under-researched and this work is badly needed. 

 The one gap that I see is in the definition of a Rehabilitation Need. How will you determine if an unmet need described in the studies reviewed is a 'rehabilitation need' or a need that falls under some remit of survivorship? The newly published WHO Package of Interventions for Rehabilitation (Malignant Neoplasms) Module 7 may prove useful as a framework for data extraction.

Is the study design appropriate for the research question?

Yes

Is the rationale for, and objectives of, the study clearly described?

Yes

Are sufficient details of the methods provided to allow replication by others?

Partly

Are the datasets clearly presented in a useable and accessible format?

Not applicable

Reviewer Expertise:

Cancer rehabilitation and survivorship

I confirm that I have read this submission and believe that I have an appropriate level of expertise to confirm that it is of an acceptable scientific standard, however I have significant reservations, as outlined above.
